# Supplementary material for: The aging transcriptome and cellular landscape of the human lung in relation to SARS-CoV-2
Source: Nat Commun. 2021 Jan 4;12:4. doi: 10.1038/s41467-020-20323-9 (PMC7782551; doi:10.1038/s41467-020-20323-9)
Supplement: Supplementary file 1 — Supplementary Information [file 41467_2020_20323_MOESM1_ESM.pdf]

Supplementary Information

The aging transcriptome and cellular landscape of the human lung in relation to SARS-CoV-2

Ryan D. Chow, Medha Majety, and Sidi Chen

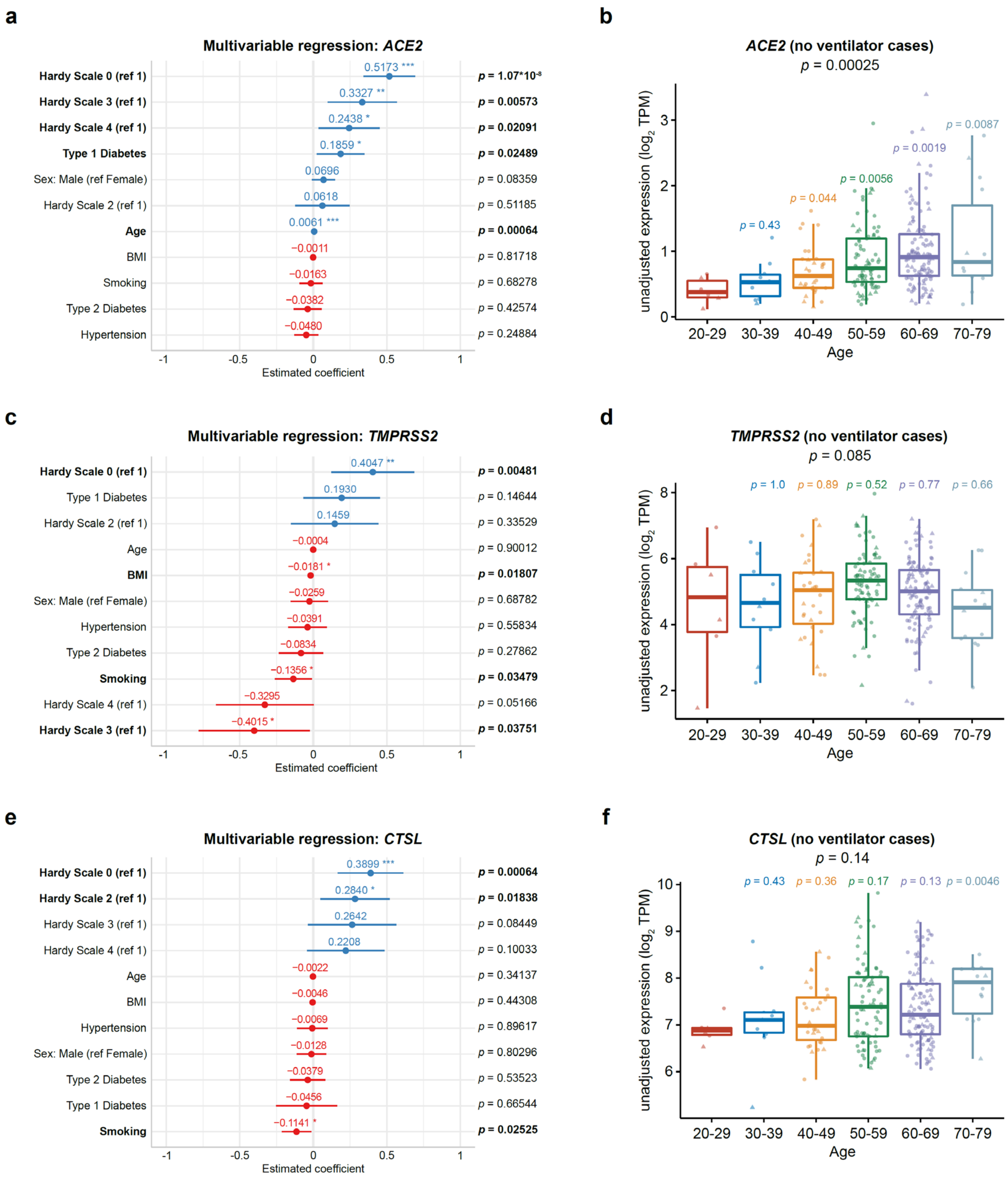

Supplementary Fig. 1: Clinical features associated with expression of SARS-CoV-2 entry factors. a,c,e. Forest plots of the multivariable regression coefficients for various clinical features in relation to the lung expression of SARS-CoV-2 entry factors *ACE2* **(a)**, *TMPRSS2* **(c)**, and *CTSL* **(e)** (n = 554 samples). A Hardy scale of 1 was used as the reference for comparisons (Hardy scale 0: on ventilator prior to death, 1: violent and fast death, 2: fast death of natural causes, 3: intermediate death, 4: slow death from chronic illness). Each point denotes the

estimated regression coefficient with error bars indicating 95% confidence intervals, and associated p-values are annotated to the right. No multiple hypothesis correction was performed. Significantly associated features are bolded and highlighted with asterisks. * $p < 0.05$, ** $p < 0.01$, *** $p < 0.001$. **b,d,f.** Tukey boxplots (interquartile range (IQR) boxes with 1.5 × IQR whiskers) detailing the expression of *ACE2* **(b)**, *TMPRSS2* **(d)**, and *CTSL* **(f)** in lung samples from donors that were not on a ventilator prior to tissue collection. Data are shown as unadjusted $\log_2$ transcripts per million (TPM). Statistical significance of the expression variation across all age groups was assessed by two-sided Kruskal-Wallis test. Pairwise comparisons of each age group to the 20-29 years-old group are also shown, color-coded by the age group (two-tailed Mann-Whitney test).

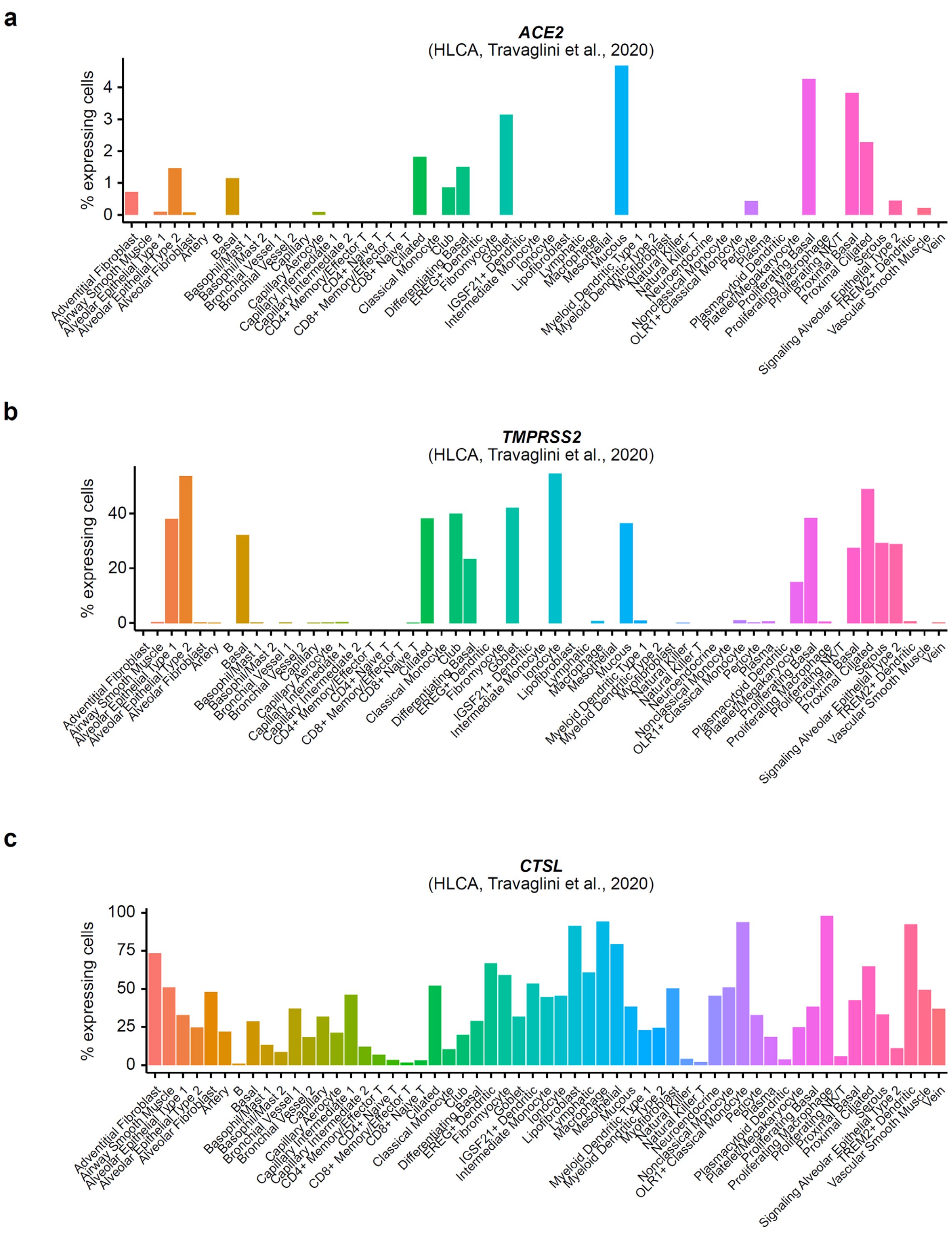

Supplementary Fig. 2: Expression of SARS-CoV-2 host factors across lung cell types (Human Lung Cell Atlas). a-c. Bar plot showing the percentage of *ACE2*-expressing cells **(a)**, *TMPRSS2*-expressing cells **(b)**, or *CTSL*-expressing cells **(c)**, grouped by cell type. Data are from the Human Lung Cell Atlas (n = 65,662 cells).

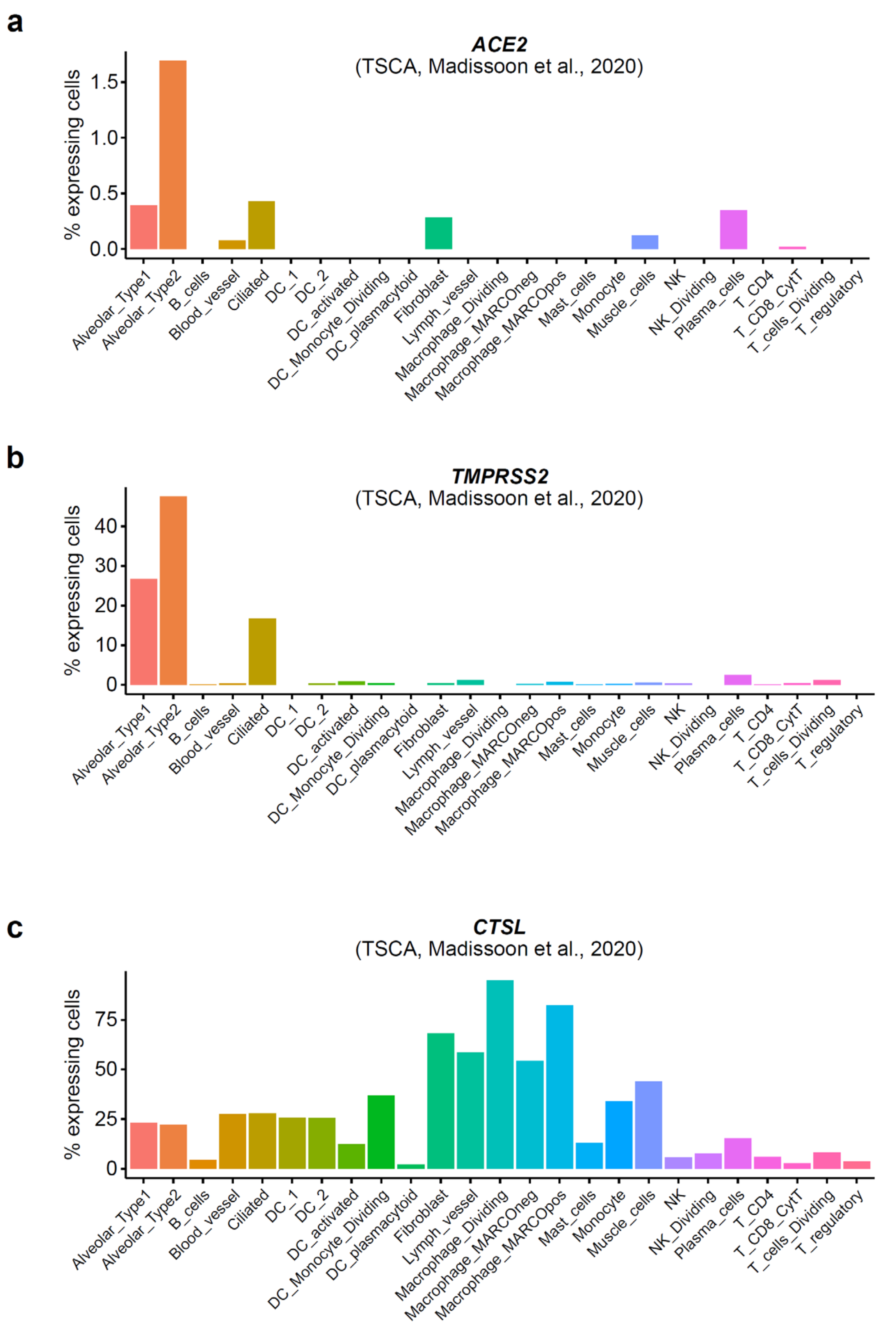

Supplementary Fig. 3: Expression of SARS-CoV-2 host factors across lung cell types (Tissue Stability Cell Atlas). a-c. Bar plot showing the percentage of *ACE2*-expressing cells **(a)**, *TMPRSS2*-expressing cells **(b)**, or *CTSL*-expressing cells **(c)**, grouped by cell type. Data are from the Tissue Stability Cell Atlas (n = 57,020 cells).

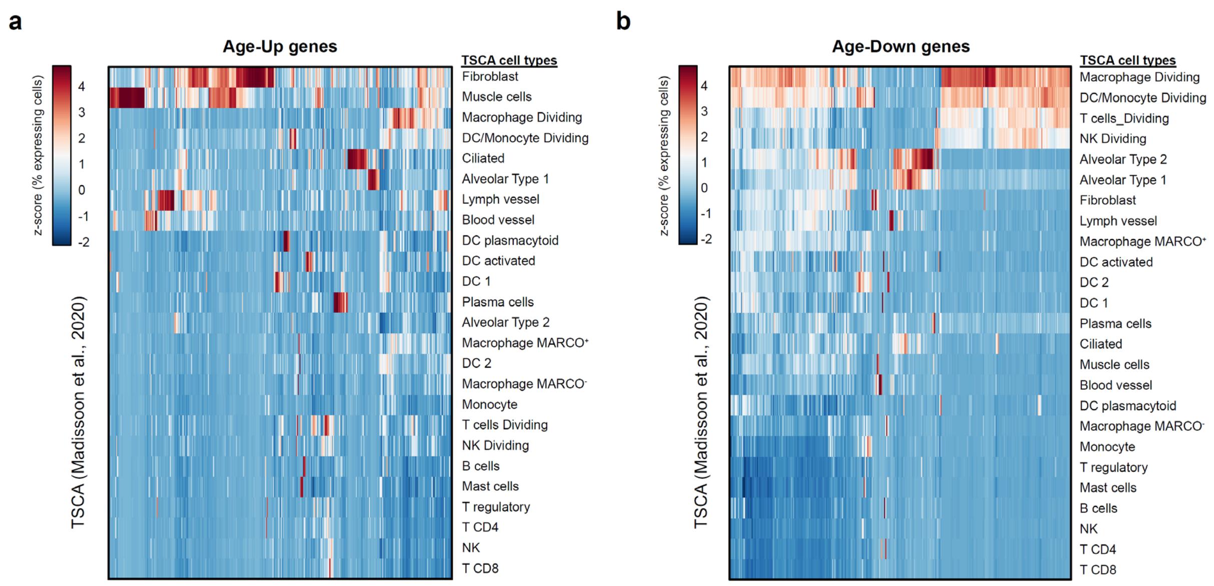

Supplementary Fig. 4: Additional cell type-specific analysis of age-associated genes. a. Heatmap showing the percentage of cells expressing each of the Age-Up genes (increasing with age), scaled by gene across the different cell types. Data are from the Tissue Stability Cell Atlas. **b.** Heatmap showing the percentage of cells expressing each of the Age-Down genes (decreasing with age), scaled by gene across the different cell types. Data are from the Tissue Stability Cell Atlas.

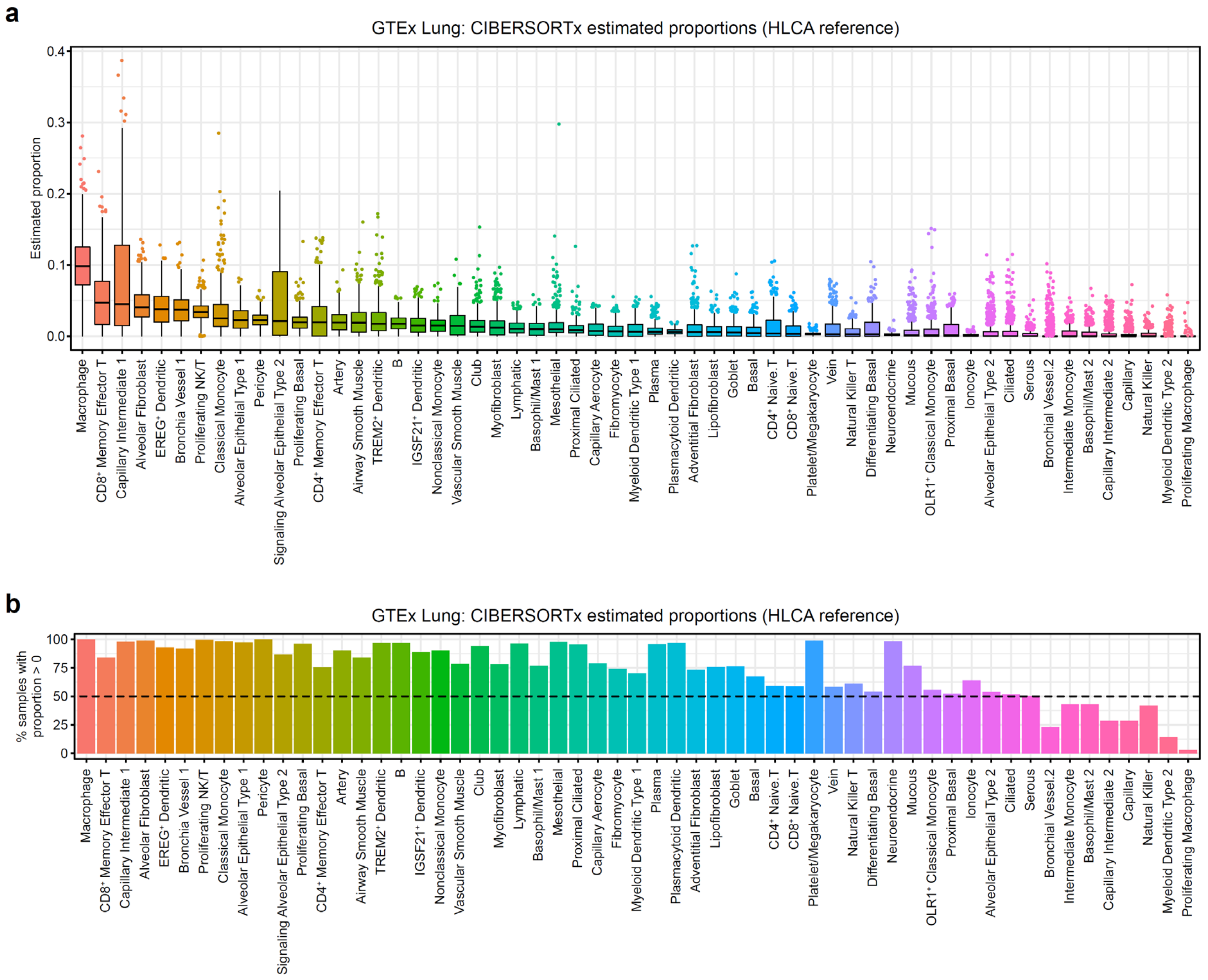

Supplementary Fig. 5: Additional analysis of lung deconvolution. a. Tukey boxplots (interquartile range (IQR) boxes with 1.5 × IQR whiskers) showing the estimated proportions of each lung cell type across all GTEx lung samples (n = 578 samples), as determined by deconvolution with CIBERSORTx (n = 57 cell types). **b.** Bar plot showing the % of samples with non-zero estimated proportions for each cell type (n = 578 samples). Cell types with non-zero proportions in < 50% of samples were excluded from further analysis (dotted line).

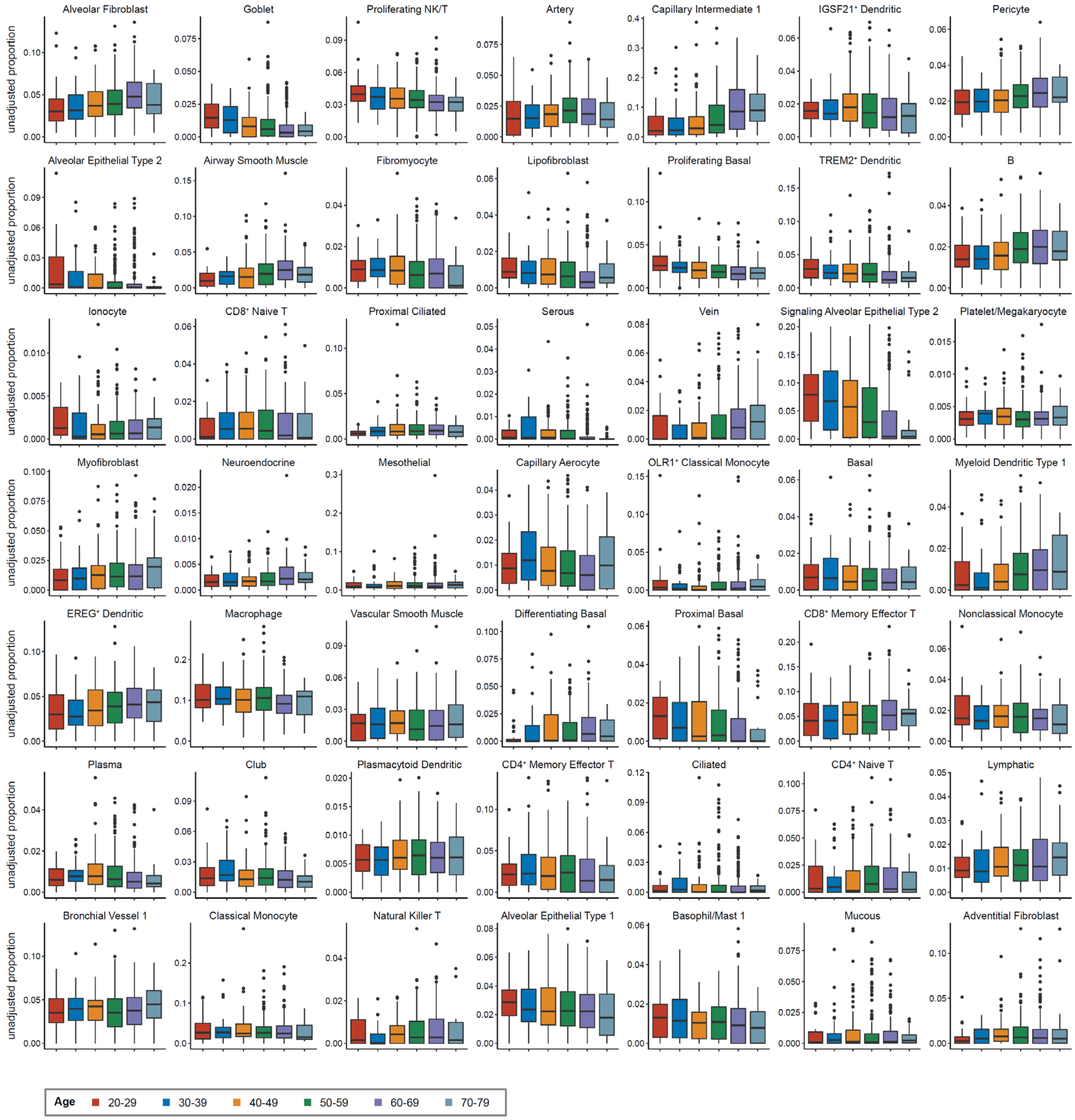

Supplementary Fig. 6: Unadjusted cell type proportions in relation to age. Tukey boxplots (interquartile range (IQR) boxes with 1.5 × IQR whiskers) showing the unadjusted estimated proportions of each lung cell type across different age groups (n = 561 samples).

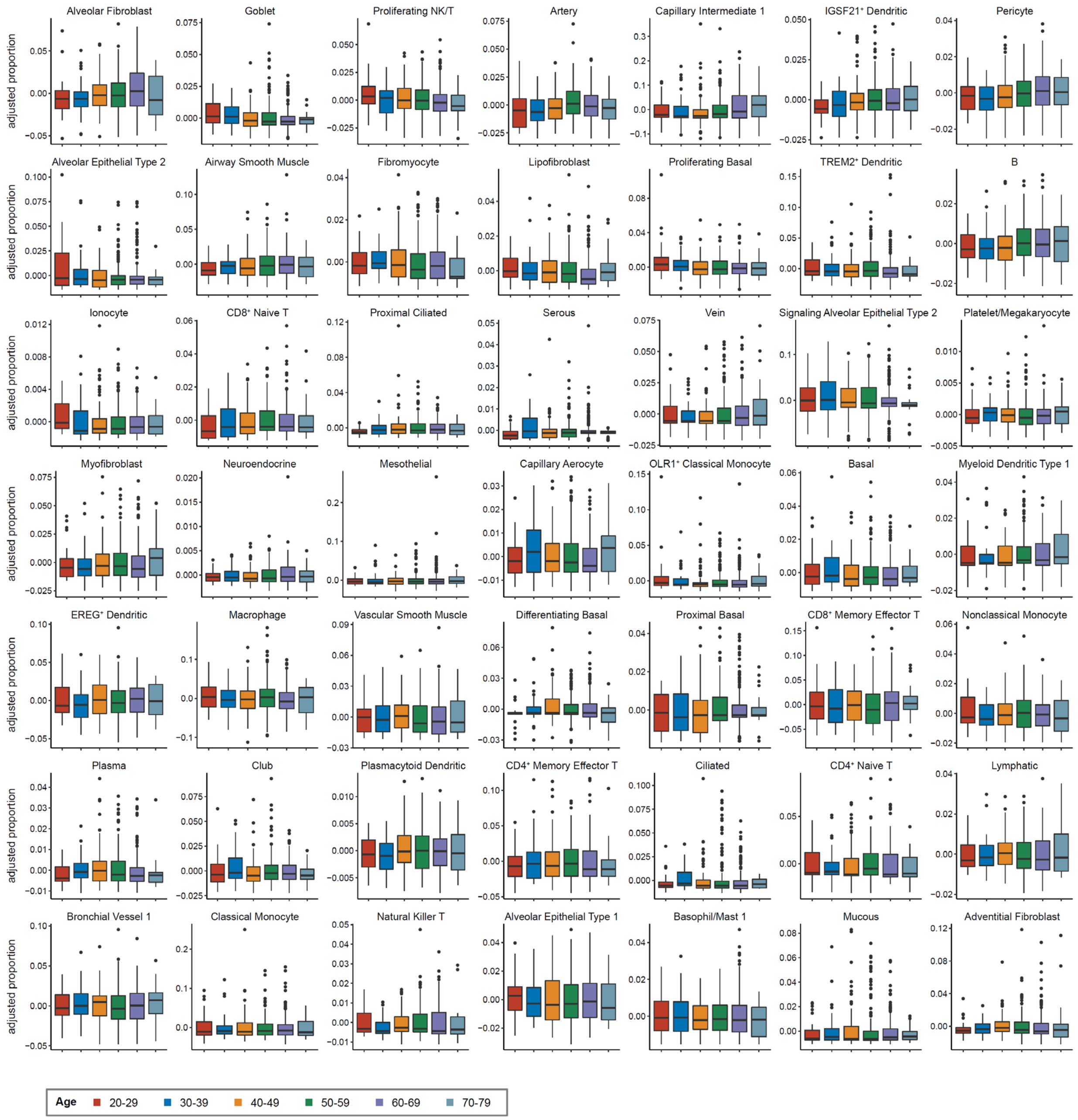

Supplementary Fig. 7: Adjusted cell type proportions in relation to age. Tukey boxplots (interquartile range (IQR) boxes with 1.5 × IQR whiskers) showing the adjusted estimated proportions of each lung cell type across different age groups (n = 561 samples). The estimated proportions were adjusted by extracting the residuals from a generalized linear model that included sex, smoking status, and Hardy scale as covariates.

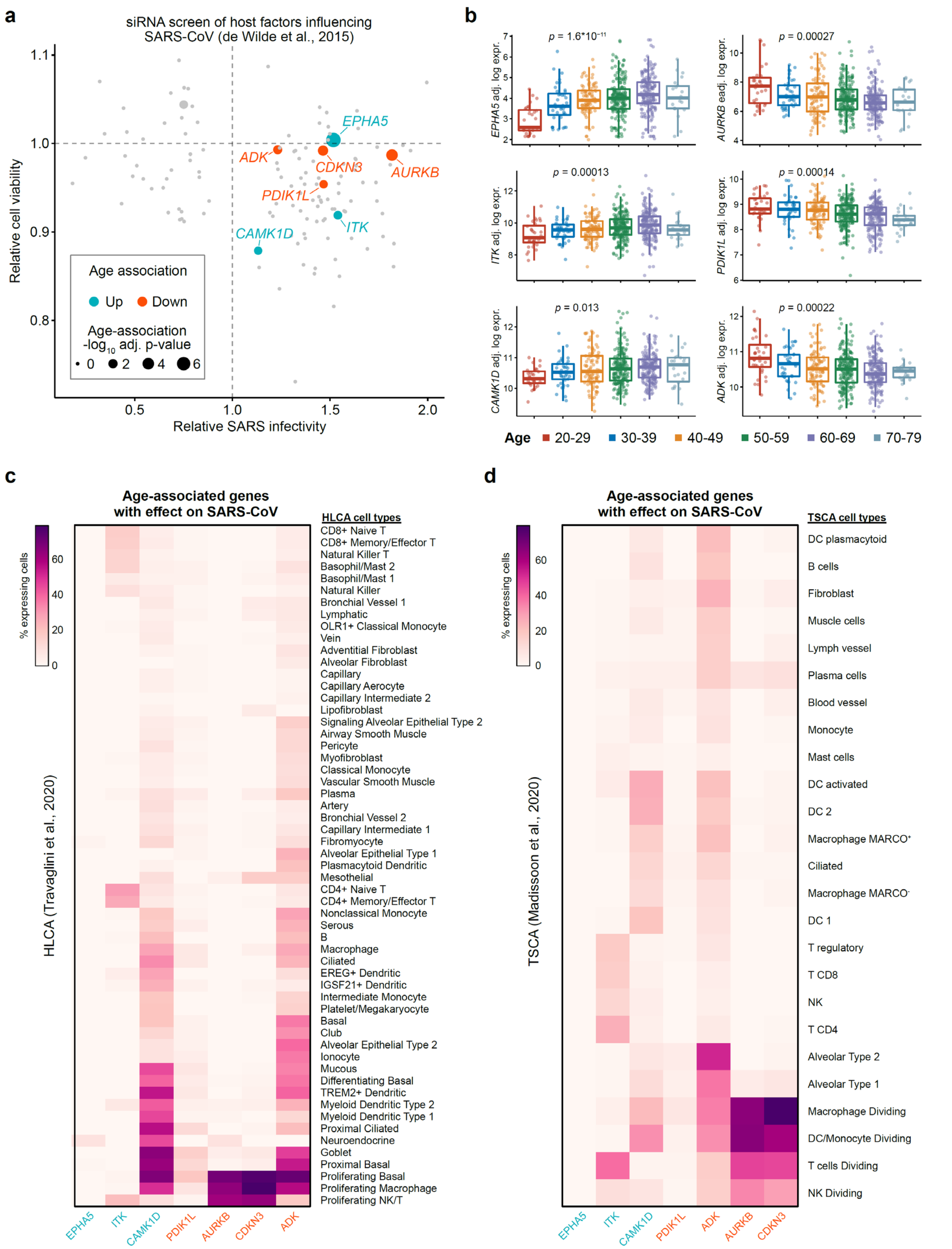

Supplementary Fig. 8: Age-associated genes in human lung influence SARS-CoV replication. a. Scatter plot of data from a published siRNA screen of host factors that influence SARS-CoV pathogenesis (from de Wilde et al., 2015). Data are shown in terms of relative

SARS-CoV infectivity and relative cell viability upon perturbation of various host factors. Values >1.0 for relative SARS-CoV infectivity suggest that knockdown of the target gene promotes infection. Values >1.0 for relative cell viability suggest that knockdown of the target gene promotes cell survival. The size of each point is scaled by the statistical significance of the association between expression of the gene and aging. Genes identified in Age-Up (increasing with age) and Age-Down (decreasing with age) are additionally color-coded (blue and orange, respectively). **b.** Tukey boxplots (interquartile range (IQR) boxes with 1.5 × IQR whiskers) detailing the expression of select age-associated genes with potential roles in SARS-CoV pathogenesis (n = 561 samples). Data are shown as log-transformed expression values, adjusted for sex, smoking status, and Hardy scale. Statistical significance of the expression variation across all age groups was assessed by Kruskal-Wallis test**. c-d.** Heatmaps showing the percentage of cells expressing each of the Age-Up genes (increasing with age, blue) and Age-Down genes (decreasing with age, orange) with an effect on SARS-CoV, as highlighted in **(a)**. Data are from the Human Lung Cell Atlas **(c)** and the Tissue Stability Cell Atlas **(d)**.

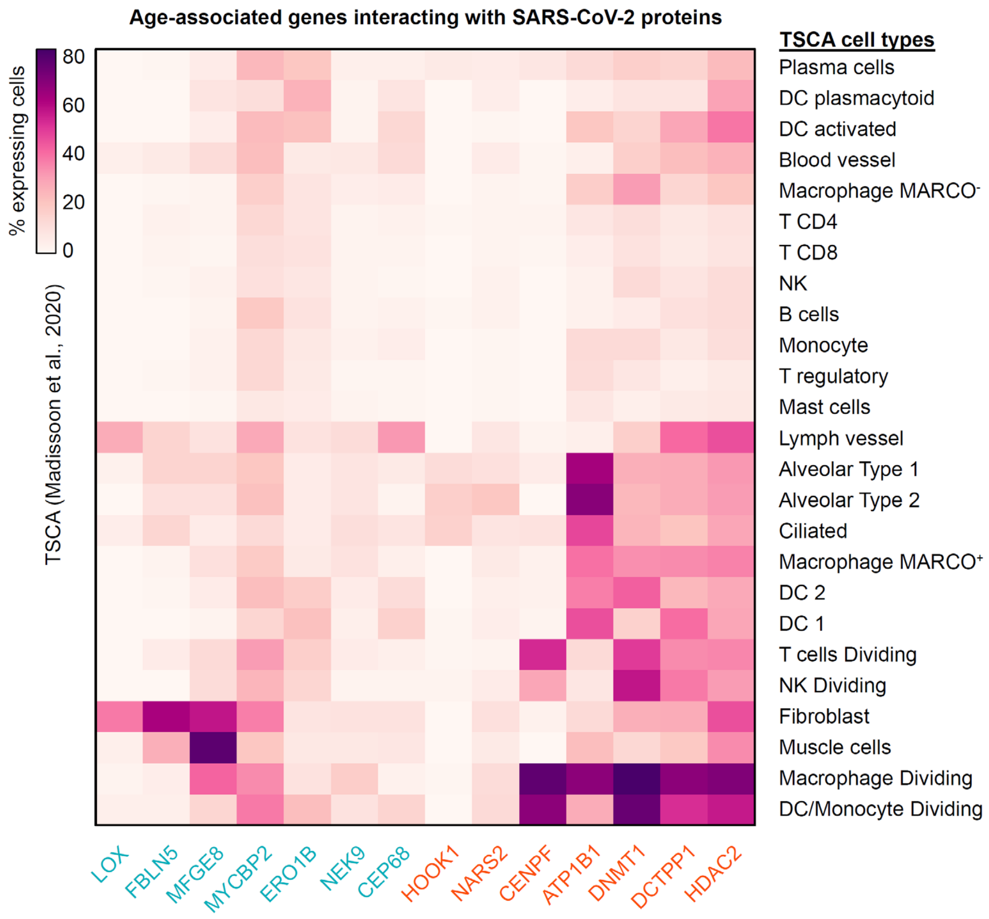

Supplementary Fig. 9: Additional cell type-specific analysis of age-associated factors that interact with SARS-CoV-2 proteins. Heatmap showing the percentage of cells expressing each of the Age-Up genes (increasing with age, blue) and Age-Down genes (decreasing with age, orange) that interact with SARS-CoV-2 proteins, as highlighted in Fig. 4b. Data are from the Tissue Stability Cell Atlas.

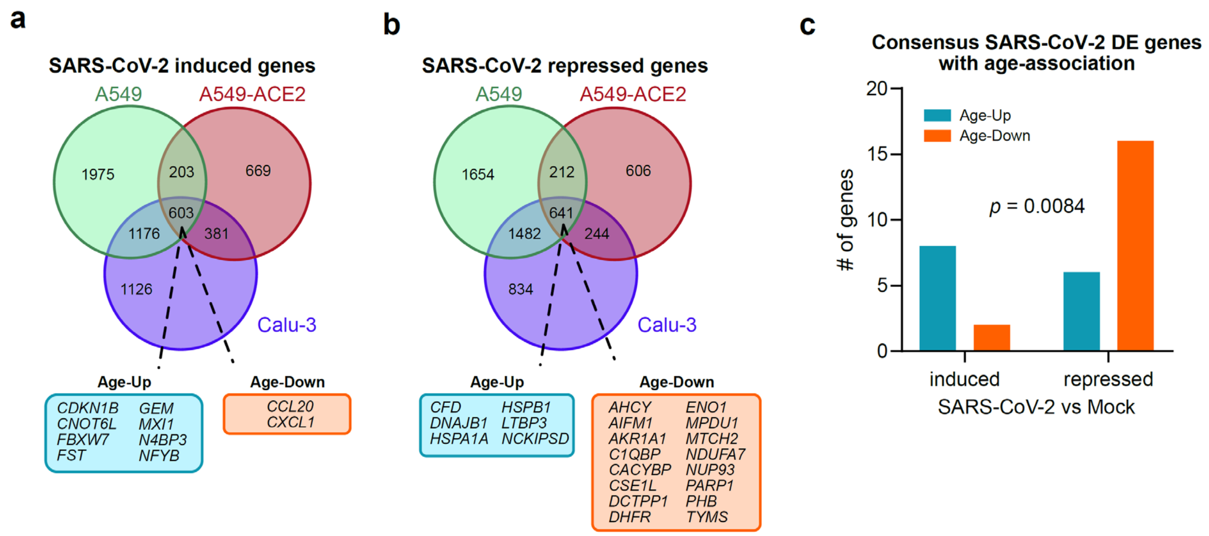

Supplementary Fig. 10: Consensus differentially expressed genes upon SARS-CoV-2 infection in vitro. a-b. Venn diagram of shared SARS-CoV-2 induced genes **(a)** or SARS-CoV-2 repressed genes **(b)** in A549 cells, A549-ACE2 cells, and Calu-3 cells. Age-associated genes lying at the intersection of all 3 datasets are detailed, classified by the directionality of age association. **c.** Characteristics of age-associated genes that are concordantly regulated by SARS-CoV-2 infection across all 3 cell lines, from **a-b**. Statistical significance of the interaction between the directionality of SARS-CoV-2 regulation (induced or repressed) and the directionality of age-association (increase or decrease with age) was assessed by two-tailed Fischer's exact test.

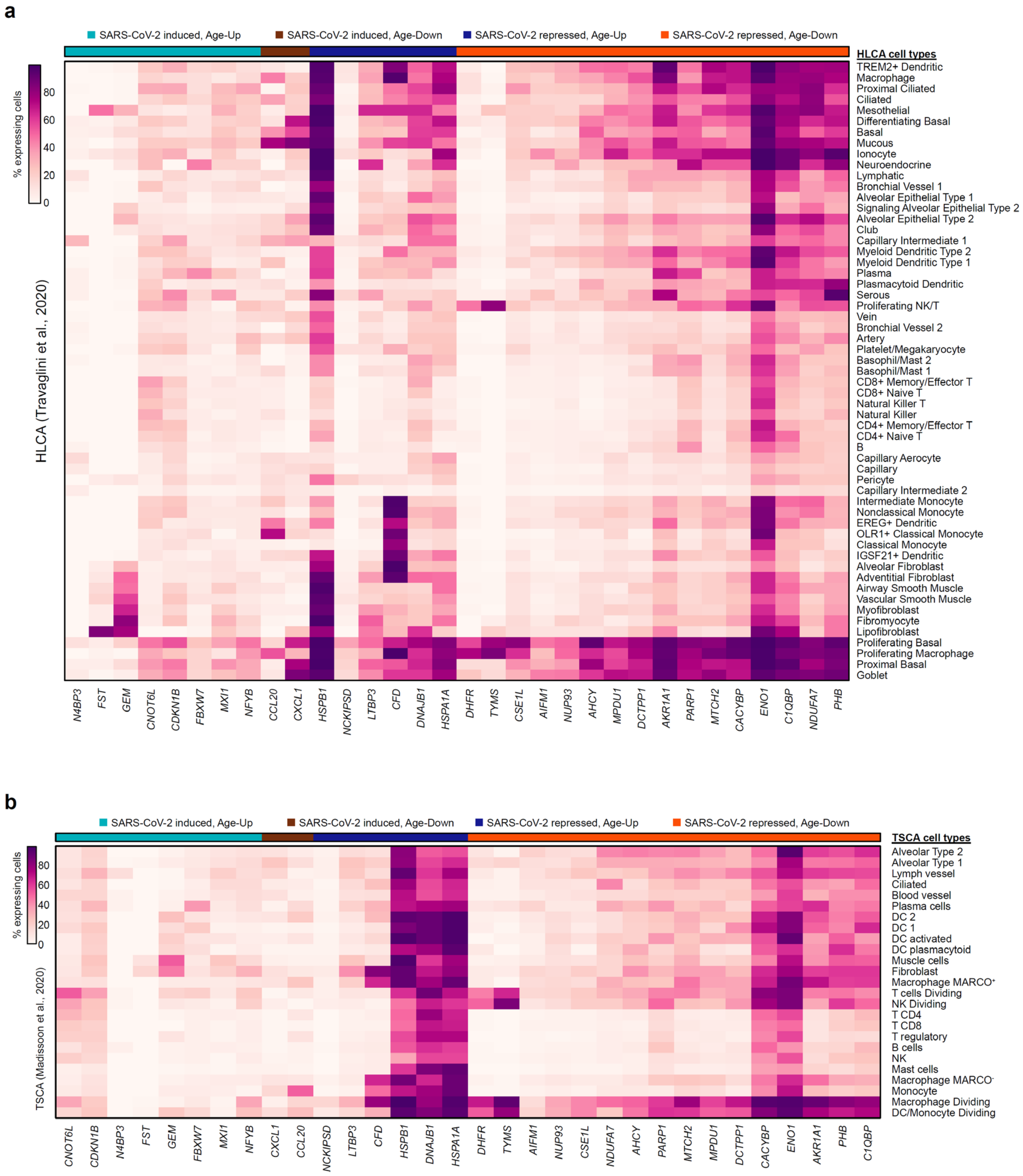

Supplementary Fig. 11: Cell type-specific analysis of age-associated factors that are differentially expressed upon SARS-CoV-2 infection in vitro. a-b. Heatmap showing the percentage of cells expressing each of the genes highlighted in Supplementary Fig. 10a-b, using data from the Human Lung Cell Atlas **(a)** or the Tissue Stability Cell Atlas **(b)**. Genes are annotated by whether they are induced or repressed by SARS-CoV-2 infection, and whether they increase (Age-Up) or decrease (Age-Down) in expression with age.

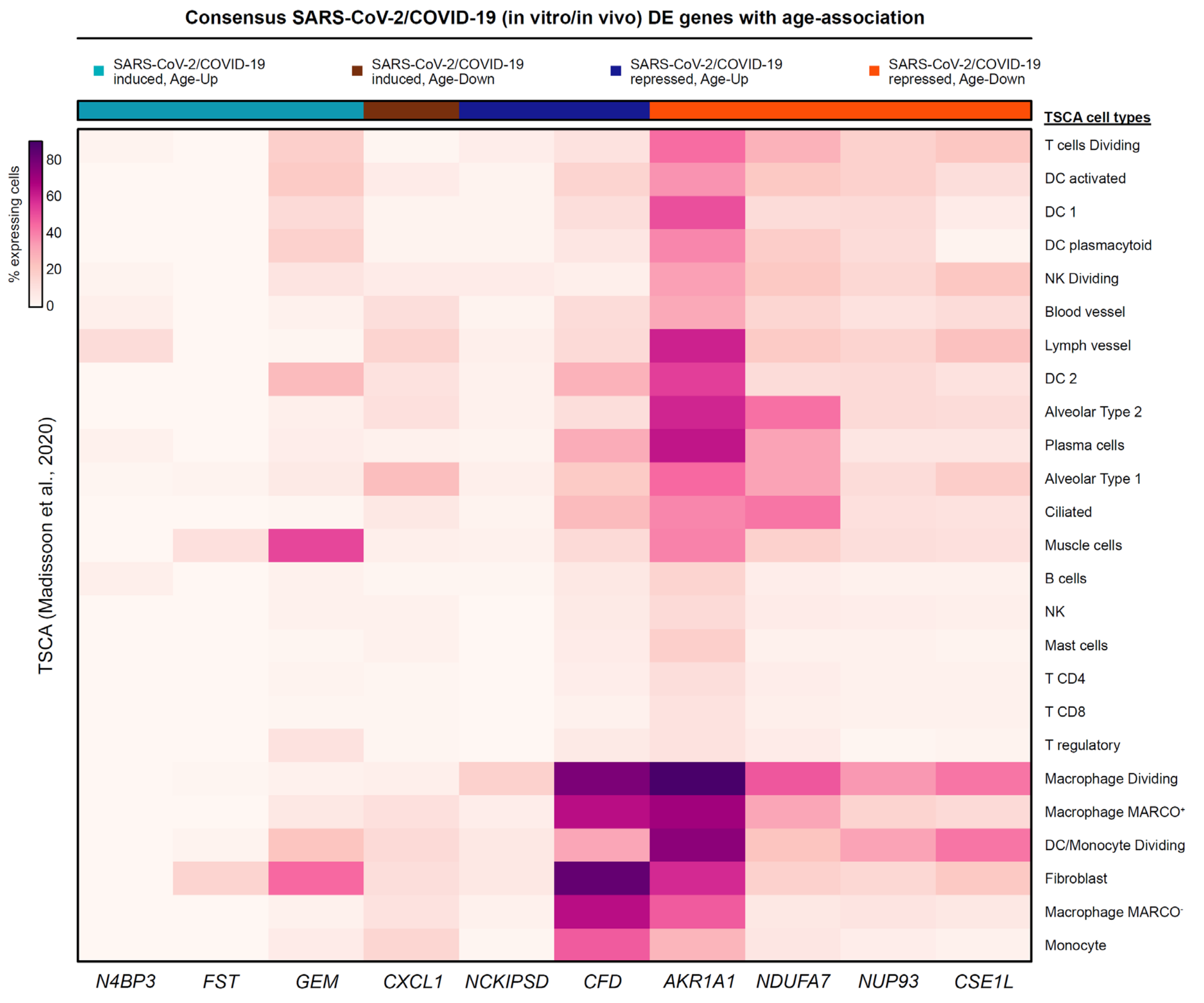

Supplementary Fig. 12: Additional cell type-specific analysis of age-associated factors that are differentially expressed in COVID-19 patients. Heatmap showing the percentage of cells expressing the consensus SARS-CoV-2/COVID-19 regulated genes that also exhibit age-associated expression, from Fig. 6e. Genes are annotated by whether they are induced or repressed by SARS-CoV-2/COVID-19, and whether they increase (Age-Up) or decrease (Age-Down) in expression with age. Data are from the Tissue Stability Cell Atlas.
