# Supplementary material for: The aging transcriptome and cellular landscape of the human lung in relation to SARS-CoV-2
Source: Nat Commun. 2021 Jan 4;12:4. doi: 10.1038/s41467-020-20323-9 (PMC7782551; doi:10.1038/s41467-020-20323-9)
Supplement: Supplementary file 3 — Description of Additional Supplementary Files [file 41467_2020_20323_MOESM3_ESM.docx]

**Description of Additional Supplementary Files**

File Name: Supplementary Data 1

Description: Demographics of donors for GTEx lung samples.

File Name: Supplementary Data 2

Description: Normalized expression matrix for SARS-CoV-2 host entry factors in human lung.

File Name: Supplementary Data 3

Description: Multivariable regression model of clinical features in relation to ACE2, TMPRSS2, and CTSL expression.

File Name: Supplementary Data 4

Description: Cell type-specific expression frequencies for SARS-CoV-2 host entry factors in human lung (Human Lung Cell Atlas).

File Name: Supplementary Data 5

Description: Cell type-specific expression frequencies for SARS-CoV-2 host entry factors in human lung (Tissue Stability Cell Atlas).

File Name: Supplementary Data 6

Description: Two-sided likelihood-ratio test table for evaluating age-associated genes, controlling for sex, smoking status, and Hardy scale.

File Name: Supplementary Data 7

Description: Final set of age-associated genes.

File Name: Supplementary Data 8

Description: DAVID analysis table for genes that increase in expression with age, with Benjamini-Hochberg multiple hypothesis adjustment.

File Name: Supplementary Data 9

Description: DAVID analysis table for genes that decrease in expression with age, with Benjamini-Hochberg multiple hypothesis adjustment.

File Name: Supplementary Data 10

Description: Cell type-specific expression frequencies for genes that increase in expression with age (Human Lung Cell Atlas).

File Name: Supplementary Data 11

Description: Cell type-specific expression frequencies for genes that increase in expression with age, transformed to z-scores (Human Lung Cell Atlas).

File Name: Supplementary Data 12

Description: Cell type-specific expression frequencies for genes that decrease in expression with age (Human Lung Cell Atlas).

File Name: Supplementary Data 13

Description: Cell type-specific expression frequencies for genes that decrease in expression with age, transformed to z-scores (Human Lung Cell Atlas).

File Name: Supplementary Data 14

Description: Cell type-specific expression frequencies for genes that increase in expression with age (Tissue Stability Cell Atlas).

File Name: Supplementary Data 15

Description: Cell type-specific expression frequencies for genes that increase in expression with age, transformed to z-scores (Tissue Stability Cell Atlas).

File Name: Supplementary Data 16

Description: Cell type-specific expression frequencies for genes that decrease in expression with age (Tissue Stability Cell Atlas).

File Name: Supplementary Data 17

Description: Cell type-specific expression frequencies for genes that decrease in expression with age, transformed to z-scores (Tissue Stability Cell Atlas).

File Name: Supplementary Data 18

Description: Estimated cell proportions from CIBERSORTx in each GTEx lung sample.

File Name: Supplementary Data 19

Description: Age-association statistics for CIBERSORTx estimated cell proportions, controlling for sex, smoking status, and Hardy scale. No multiple hypothesis correction.

File Name: Supplementary Data 20

Description: Annotation of a prior SARS-CoV siRNA screen (de Wilde et al., 2015) with age-association statistics from this study. No multiple hypothesis correction.

File Name: Supplementary Data 21

Description: Annotation of the SARS-CoV-2:Human protein interactome (Gordon et al., 2020) with age-association statistics from this study.

File Name: Supplementary Data 22

Description: Differential expression analysis in A549 cells, infected with SARS-CoV-2 vs mock control, with age-association annotations. Multiple hypothesis correction by Benjamini-Hochberg method.

File Name: Supplementary Data 23

Description: Differential expression analysis in A549-ACE2 cells, infected with SARS-CoV-2 vs mock control, with age-association annotations. Multiple hypothesis correction by Benjamini-Hochberg method.

File Name: Supplementary Data 24

Description: Differential expression analysis in Calu-3 cells, infected with SARS-CoV-2 vs mock control, with age-association annotations. Multiple hypothesis correction by Benjamini-Hochberg method.

File Name: Supplementary Data 25

Description: Characteristics of age-associated genes that are regulated by SARS-CoV-2 infection.

File Name: Supplementary Data 26

Description: Consensus differentially expressed genes upon SARS-CoV-2 infection, intersected with age-associated genes.

File Name: Supplementary Data 27

Description: Differential expression analysis comparing patients with severe COVID19 vs. healthy controls, with age-association annotations. Multiple hypothesis correction by Bonferroni method.

File Name: Supplementary Data 28

Description: Characteristics of age-associated genes that are regulated by COVID-19.

File Name: Supplementary Data 29

Description: Common differentially expressed genes upon SARS-CoV-2 infection in vitro and in severe COVID-19 patients, intersected with age-associated genes.
