# Supplementary material for: The aging transcriptome and cellular landscape of the human lung in relation to SARS-CoV-2
Source: Nat Commun. 2021 Jan 4;12:4. doi: 10.1038/s41467-020-20323-9 (PMC7782551; doi:10.1038/s41467-020-20323-9)
Supplement: Supplementary file 33 — Reporting Summary [file 41467_2020_20323_MOESM33_ESM.pdf]

nature research

Corresponding author(s): Sidi Chen

Last updated by author(s): Oct 23, 2020

Reporting Summary

Nature Research wishes to improve the reproducibility of the work that we publish. This form provides structure for consistency and transparency in reporting. For further information on Nature Research policies, see Authors & Referees and the Editorial Policy Checklist.

Statistics

For all statistical analyses, confirm that the following items are present in the figure legend, table legend, main text, or Methods section.

n/a	Confirmed	
☐	☒	The exact sample size (n) for each experimental group/condition, given as a discrete number and unit of measurement
☒	☐	A statement on whether measurements were taken from distinct samples or whether the same sample was measured repeatedly
☐	☒	The statistical test(s) used AND whether they are one- or two-sided<br>*Only common tests should be described solely by name; describe more complex techniques in the Methods section.*
☐	☒	A description of all covariates tested
☐	☒	A description of any assumptions or corrections, such as tests of normality and adjustment for multiple comparisons
☐	☒	A full description of the statistical parameters including central tendency (e.g. means) or other basic estimates (e.g. regression coefficient) AND variation (e.g. standard deviation) or associated estimates of uncertainty (e.g. confidence intervals)
☐	☒	For null hypothesis testing, the test statistic (e.g. F, t, r) with confidence intervals, effect sizes, degrees of freedom and P value noted<br>*Give P values as exact values whenever suitable.*
☒	☐	For Bayesian analysis, information on the choice of priors and Markov chain Monte Carlo settings
☒	☐	For hierarchical and complex designs, identification of the appropriate level for tests and full reporting of outcomes
☒	☐	Estimates of effect sizes (e.g. Cohen's d, Pearson's r), indicating how they were calculated

Our web collection on statistics for biologists contains articles on many of the points above.

Software and code

Policy information about availability of computer code

Data collection	No software was used for data collection.
Data analysis	Clinical annotations for age, sex, obesity, hypertension, type 1 diabetes, type 2 diabetes, Hardy scale, and smoking history were compiled from the controlled access GTEx metadata. A Hardy scale of 1 was used as the reference for comparisons (Hardy scale 0: on ventilator prior to death, 1: violent and fast death, 2: fast death of natural causes, 3: intermediate death, 4: slow death from chronic illness). With these annotations, a multivariable linear regression model was utilized to assess whether different clinical features were associated with the log-transformed expression of SARS-CoV-2 entry factors. The resulting estimated regression coefficients were visualized as forest plots with 95% confidence intervals.<br><br>To identify age-associated genes, the RNA-seq raw count matrix was analyzed by DESeq2 (v1.24.0), using the likelihood ratio test (LRT). Sex, smoking status, and Hardy scale were included in the LRT model to control for these factors. Donor ages were binned into decades (e.g. 20-29, 30-39, 40-49, 50-59, 60-69, 70-79) for analysis. Age-associated genes were determined at a significance threshold of adjusted $p < 0.05$. Genes passing the significance threshold were then scaled to z-scores and clustered using the degPatterns function from the R package DEGreport (v1.20.0). Gene clusters with progressive and consistent trends with age were retained for downstream analysis. Gene ontology and pathway enrichment analysis was performed using DAVID (v6.8) (https://david.ncifcrf.gov/), separating the age-associated genes into two primary clusters (increasing or decreasing with age).<br><br>For visualization of unadjusted RNA-seq expression data, the TPM values were log2 transformed and plotted in R (v3.6.1). For visualization of adjusted expression levels, the raw counts were first processed by variance-stabilizing transformation with DESeq2, followed by statistical adjustment for sex, smoking status, and Hardy scale using the removeBatchEffect function in limma (v3.45). All boxplots are Tukey boxplots, with interquartile range (IQR) boxes and 1.5×IQR whiskers. Pairwise statistical comparisons in the plots were assessed by two-tailed Mann-Whitney test, while statistical comparisons across all age groups were performed by Kruskal-Wallis test. We note that the identification of age-associated genes was purely determined through the DESeq2 likelihood ratio test described above; the Mann-Whitney or Kruskal-Wallis statistics shown on the plots are solely for confirmatory purposes.

scRNA-seq data were analyzed in R (v3.6.1) using Seurat (v3.2) and custom scripts. Of the 782 age-associated genes identified from GTEx bulk transcriptomes, 712 genes were matched in the Tissue Stability Cell Atlas dataset and 683 genes were matched in the Human Lung Cell Atlas dataset. To determine the percentage of cells expressing a given gene, the expression matrices were converted to binary matrices by setting a threshold of expression > 0. Cell type-specific expression frequencies for each gene were then calculated using the provided cell type annotations. To identify genes preferentially expressed in a specific cell type, we further scaled the expression frequencies in R to obtain z-scores. Data were visualized in R using the NMF package (v0.23).

To infer the cellular composition of each bulk lung transcriptome, we used the CIBERSORTx algorithm. We provided the Human Lung Cell Atlas dataset as a reference to calculate estimated cell type proportions in each lung sample with S-mode batch correction. The resultant cell type proportions were analyzed in R. Cell types with estimated proportions of "0" in >50% of samples were filtered out prior to further analysis. Statistical significance of age-association was assessed by an ordinal logistic regression model, a generalization of the non-parametric Kruskal-Wallis test that allows for multifactorial designs. Sex, smoking status, and Hardy scale were included in the regression model to pinpoint the cell types that are specifically altered with aging. The estimated coefficients were visualized as a forest plot with 95% confidence intervals.

To assess whether any age-associated genes affect host responses to SARS-CoV (a coronavirus related to SARS-CoV-2), we analyzed the data from a published siRNA screen of host factors influencing SARS-CoV (Data Set S1 in the publication; accessed on March 20, 2020). For data visualization, each point corresponding to a target gene was size-scaled and color-coded according to the age-association statistical analyses described above.

To assess whether any lung age-associated genes encode proteins that interact with the SARS-CoV-2 proteome, we compiled the data from a preprint manuscript detailing the human host factors that interact with 27 different proteins in the SARS-CoV-2 proteome (accessed on March 23, 2020).

To assess whether the expression of lung age-associated genes is influenced by SARS-CoV-2 infection, we utilized the data from a recent study detailing the transcriptional response to SARS-CoV-2 infection, from the Gene Expression Omnibus (GSE147507) (accessed on April 13, 2020). Differentially expressed genes were determined using the Wald test in DESeq2 (v1.24.0) comparing SARS-CoV-2 infected cells to batch-matched mock controls, with a significance threshold of adjusted $p < 0.05$. Of the 782 age-associated genes, 641 genes were matched to the RNA-seq dataset. Statistical significance of overlaps between the gene sets was assessed by two-tailed hypergeometric test, assuming 21,797 total genes as annotated in the RNA-seq dataset and 641 age-associated genes. Statistical significance of the association between the directionality of SARS-CoV-2 regulation and the directionality of age-association was assessed by two-tailed Fischer's exact test.

To assess whether the expression of lung age-associated genes is affected in patients with severe COVID-19, we utilized the data from a recent study detailing the transcriptomes of bronchioalveolar lavage fluid cells from patients with COVID-19 74, from the Gene Expression Omnibus (GSE145926) (accessed on May 14, 2020). Cells were filtered in a similar manner as previously described by the study authors (unique RNA species ≥ 200 and ≤ 6,000, mitochondrial reads ≤ 10%, and UMI ≥ 1,000), The single cell transcriptomes were collapsed into pseudo-bulk profiles by summing the read counts for all the cells from each donor. Differentially expressed genes were then determined using the Wald test in DESeq2 (v1.24.0) 32 comparing patients with severe COVID-19 to healthy controls, with a significance threshold of adjusted $p < 0.05$.

Of the 782 age-associated genes, 675 genes were matched to the scRNA-seq pseudo-bulk dataset. Statistical significance of overlaps between the gene sets was assessed by hypergeometric test, assuming 33,540 total genes as annotated in the scRNA-seq dataset and 675 age-associated genes. Statistical significance of the association between the directionality of COVID-19 regulation and the directionality of age-association was assessed by two-tailed Fischer's exact test.

All analysis code has deposited to GitHub: https://github.com/rdchow/agingLung-COVID

For manuscripts utilizing custom algorithms or software that are central to the research but not yet described in published literature, software must be made available to editors/reviewers. We strongly encourage code deposition in a community repository (e.g. GitHub). See the Nature Research guidelines for submitting code & software for further information.

Data

Policy information about availability of data

All manuscripts must include a data availability statement. This statement should provide the following information, where applicable:

- Accession codes, unique identifiers, or web links for publicly available datasets
- A list of figures that have associated raw data
- A description of any restrictions on data availability

All relevant processed data generated during this study are included in this article and its supplementary information files. Source data for this paper are provided in the Supplementary Data. Raw data are from various sources as described above. Accession codes: GTEx (phs000424.v8.p2), Human Lung Cell Atlas (#syn21041850), Tissue Stability Cell Atlas (PRJEB31843), SARS-CoV-2 infection in vitro (GSE147507), COVID-19 patients (GSE145926). All data related to this study are freely available from the links provided in the Methods section or from the corresponding author upon request, with the exception of detailed clinical annotations on the GTEx cohort that are under controlled access.

Field-specific reporting

Please select the one below that is the best fit for your research. If you are not sure, read the appropriate sections before making your selection.

☒ Life sciences ☐ Behavioural & social sciences ☐ Ecological, evolutionary & environmental sciences

For a reference copy of the document with all sections, see nature.com/documents/nr-reporting-summary-flat.pdf

Life sciences study design

All studies must disclose on these points even when the disclosure is negative.

Sample size	No specific calculations were performed to predetermine sample size. As this study was based on analysis of existing datasets, the sample sizes were predetermined by the original study authors, and we did not exclude any data. These sample sizes were sufficient to derive statistical conclusions in the original publications and other analogous studies using these datasets; consequently, we determined that the sample sizes of these datasets were sufficient for our own statistical analyses.
Data exclusions	No data were excluded.
Replication	Analyses are based on large datasets with several independent biological replicates. While these datasets are largely unique resources and are thus not feasible to replicate with independent datasets, we did successfully replicate our analysis of the scRNA-seq data using two distinct lung cell atlases (HLCA and TSCA). We did not perform any experiments for this study, and as such, there were no attempts at experimental replication.
Randomization	Randomization is not applicable to this bioinformatics study, as all available data were used in aggregate for analysis. We always considered relevant covariates (sex, Hardy scale, smoking status) when deriving age-associated signatures. As all data were used in aggregate for statistical analysis, there was no randomization involved in allocating specific subgroups. Additionally, as described above, no experiments were performed for this study, and consequently, randomization was not involved in any experimental setups.
Blinding	Blinding was not applicable to this study, as all of the data are publicly available and the analyses were designed to explicitly study the effects of a specific covariate (age) in relation to other variables. The analyses were performed on samples with generic identifiers, but the investigators always had access to the specific variables in question.

Reporting for specific materials, systems and methods

We require information from authors about some types of materials, experimental systems and methods used in many studies. Here, indicate whether each material, system or method listed is relevant to your study. If you are not sure if a list item applies to your research, read the appropriate section before selecting a response.

Materials & experimental systems

n/a	Involved in the study	
☒	☐	Antibodies
☒	☐	Eukaryotic cell lines
☒	☐	Palaeontology
☒	☐	Animals and other organisms
☒	☐	Human research participants
☒	☐	Clinical data

Methods

n/a	Involved in the study	
☒	☐	ChIP-seq
☒	☐	Flow cytometry
☒	☐	MRI-based neuroimaging
